# Value of high-output pace-mapping of the right phrenic nerve for enabling safe radiofrequency ablation of atrial fibrillation: insights from three-dimensional computed tomography segmentation

**DOI:** 10.1093/europace/euae207

**Published:** 2024-07-31

**Authors:** Fabien Squara, Gregory Supple, Ioan Liuba, Michal Wasiak, Erica Zado, Benoit Desjardins, Francis E Marchlinski

**Affiliations:** Department of Cardiology, Pasteur University Hospital, 30 avenue de la Voie Romaine, 06000 Nice, France; Department of Cardiac Electrophysiology, Hospital of the University of Pennsylvania, 3400 Spruce Street, Philadelphia, PA 19104, USA; Department of Cardiac Electrophysiology, Hospital of the University of Pennsylvania, 3400 Spruce Street, Philadelphia, PA 19104, USA; Department of Cardiac Electrophysiology, Hospital of the University of Pennsylvania, 3400 Spruce Street, Philadelphia, PA 19104, USA; Department of Cardiac Electrophysiology, Hospital of the University of Pennsylvania, 3400 Spruce Street, Philadelphia, PA 19104, USA; Department of Cardiac Electrophysiology, Hospital of the University of Pennsylvania, 3400 Spruce Street, Philadelphia, PA 19104, USA; Department of Radiology, Hospital of the University of Pennsylvania, 3400 Spruce Street, Philadelphia, PA 19104, USA; Department of Cardiac Electrophysiology, Hospital of the University of Pennsylvania, 3400 Spruce Street, Philadelphia, PA 19104, USA

**Keywords:** Atrial fibrillation, Ablation, Radiofrequency, Pacing, Right phrenic nerve, CT scan, Electroanatomical mapping

## Abstract

**Aims:**

Right phrenic nerve (RPN) injury is a disabling but uncommon complication of atrial fibrillation (AF) radiofrequency ablation. Pace-mapping is widely used to infer RPN’s course, for limiting the risk of palsy by avoiding ablation at capture sites. However, information is lacking regarding the distance between the endocardial sites of capture and the actual anatomic RPN location. We aimed at determining the distance between endocardial sites of capture and anatomic CT location of the RPN, depending on the capture threshold.

**Methods and results:**

In consecutive patients undergoing AF radiofrequency ablation, we defined the course of the RPN on the electroanatomical map with high-output pacing at up to 50 mA/2 ms, and assessed RPN capture threshold (RPN-t). The true anatomic course of the RPN was delineated and segmented using CT scan, then merged with the electroanatomical map. The distance between pacing sites and the RPN was assessed. In 45 patients, 1033 pacing sites were analysed. Distances from pacing sites to RPN ranged from 7.5 ± 3.0 mm (min 1) when RPN-t was ≤10 mA to 19.2 ± 6.5 mm (min 9.4) in cases of non-capture at 50 mA. A distance to the phrenic nerve > 10 mm was predicted by RPN-t with a ROC curve area of 0.846 [0.821–0.870] (*P* < 0.001), with Se = 80.8% and Sp = 77.5% if RPN-t > 20 mA, Se = 68.0% and Sp = 91.6% if RPN-t > 30 mA, and Se = 42.4% and Sp = 97.6% if non-capture at 50 mA.

**Conclusion:**

These data emphasize the utility of high-output pace-mapping of the RPN. Non-capture at 50 mA/2 ms demonstrated very high specificity for predicting a distance to the RPN > 10 mm, ensuring safe radiofrequency delivery.

## Graphical Abstract

**Graphical Abstract:**
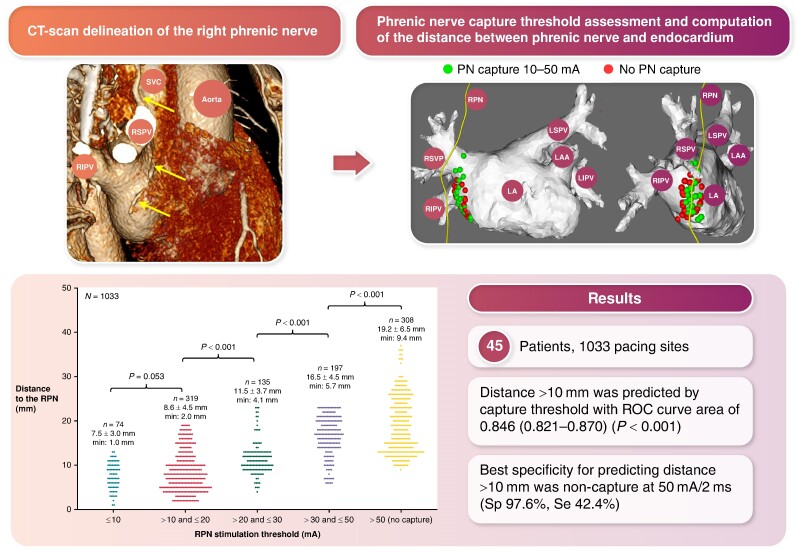

**What’s new?**

- Pace-mapping of the right phrenic nerve is widely used to infer its position during atrial fibrillation radiofrequency ablation for avoiding nerve injury; however, the actual distance between pacing sites and the nerve is unknown.
- We assessed the distance between endocardial pace-mapping sites and the true location of the right phrenic nerve delineated using CT scan, with regard to the capture threshold.
- Only non-capture at 50 mA/2 ms provided high enough specificity for predicting a distance > 10 mm, ensuring safe radiofrequency delivery.

## Introduction

The right phrenic nerve (RPN) runs vertically inside the right pericardiophrenic bundle (RPB) on the surface of the pericardium, antero-laterally to the superior vena cava (SVC), then posteriorly as it approaches the SVC-right atrium (RA) junction and the right superior pulmonary vein (RSPV), with whom it has very close anatomical relationship.^1^

Although less frequent than in the setting of balloon cryoablation of atrial fibrillation (AF),^2^ RPN injury remains a potential disabling complication following RSPV or SVC radiofrequency (RF) ablation, with an estimated prevalence ranging from 0.48% to 2.1%.^3,4^ For mitigating the risk of RPN injury during RF at these sites, preventive measures are usually performed such as upstream pacing of the RPN during RF application for monitoring diaphragm contraction, and delineation of the RPN using pace-mapping in order to avoid RF application along its course.^5,6^ However, there is a considerable heterogeneity amongst the pacing protocols used, and data are lacking about the actual distance between the atrial endocardium and the RPN when stimulated by endocardial pacing. Since the size of the virtual electrode is dependent on the output intensity,^7,8^ it is likely that the size of the critical virtual electrode capturing the RPN, i.e. the pacing threshold of the RPN, could predict the distance between the endocardium and the RPN. This might provide important information on the distance between the RPN and ablation sites for enabling safe radiofrequency delivery.

It has previously been established that the RPN can accurately been seen in most patients on CT scan, either directly^9^ or by visualization of the right pericardiophrenic vessels.^10,11^ Therefore, in this study, we aimed at determining the distance between endocardial pacing sites and the true anatomical course of the RPN using CT scan, depending on the capture threshold.

## Methods

### Patient selection

Consecutive patients undergoing first time AF RF ablation at the Hospital of the University of Pennsylvania were prospectively enrolled in the study. Patient demographics were recorded, including age, gender, comorbidities, and type of AF.

### AF ablation procedure

All procedures followed the institutional guidelines of the University of Pennsylvania Health System (UPHS), and all patients signed a written informed consent document. This work complies with the Declaration of Helsinki. Antiarrhythmic medications were discontinued five half-lives before the procedure, except amiodarone that was discontinued for at least 2 weeks. Our AF ablation approach has been previously described. Briefly, under general anaesthesia without muscle paralytics, decapolar catheters were placed in the coronary sinus (CS) and the posterior right atrium (RA). Double transseptal punctures were performed through which the ablation and decapolar circular mapping catheters (adjustable 15–25 mm circumference) were advanced into the left atrium (LA). Electroanatomical mapping was performed using CARTO (Biosense Webster, Diamond Bar, CA) or Ensite (Abbott, St Paul, MN). The mitral valve was carefully defined as were the individual PVs. CT-segmented LA anatomy was merged with the electroanatomical mapping shell.

The ablation strategy consisted of pulmonary vein (PV) isolation using two wide antral circumferential radiofrequency ablations across ipsilateral PVs and focal ablation of non-PV triggers. The standardized trigger protocol included cardioversion of induced or spontaneous AF and infusion of up to 20 μg/min isoproterenol at increments of 3, 6, 12, and 20 μg after PV isolation achieved. The procedural endpoint was PV isolation with confirmed entrance and exit block, and elimination of all non-PV triggers resulting in AF. Impedance controlled point-by-point radiofrequency (RF) ablation lesions were delivered, with RF power ≤ 40 W and temperature ≤ 42°C. Power and lesion duration were decreased over the posterior aspect of the PVs with careful attention to oesophageal temperature. At the end of the procedure, adenosine was administered intravenously, 12–18 mg, to assess for dormant LA-PV connections.

### Pace-mapping of the right phrenic nerve

As part of the standard of care at the UPHS, detailed pace-mapping of the RPN was performed along the septal part of the right PVs, ±along the posterior/lateral part of the SVC and the RA when AF triggers were documented in the SVC or at the crista terminalis. High-output pacing at 50 mA and 2 ms pulse width was first performed at these sites (Bloom® electrophysiology stimulator, Fischer Medical, Wheat Ridge, CO) using the ablation catheter with confirmed stable contact > 7 g, with assessment of RPN stimulation by palpating diaphragm contraction and visualizing diaphragm motion in fluoroscopy. When the RPN was stimulated, the capture threshold was then approximated by differential output pacing at 10, 20, and 30 mA with unchanged pulse width of 2 ms. More detailed threshold assessment could be performed at the operator’s discretion. Of note, the pulse width for reaching the phrenic nerve rheobase has been previously determined to be around 1.5 ms;^12,13^ thus, further increasing the pulse width above 2 ms would probably not affect RPN capture threshold. Right phrenic nerve capture sites were tagged on the electroanatomic system, and labelled with the capture threshold. Non-capture sites at 50 mA/2 ms were also recorded. Ultimately, all pacing sites were registered depending on the RPN threshold in the following categories: ≤10 mA, >10 and ≤20 mA, >20 and ≤30 mA, >30 and ≤50 mA, and >50 mA (no RPN capture at maximum output).

RF applications were only performed at non-capture sites, implying displacement of the planned ablation lines when necessary. Right phrenic nerve function was carefully monitored during RF applications close to the course of the RPN, by upstream stimulation of the RPN with monitoring of diaphragm contraction. In case of decrease of diaphragm motion during ablation, RF application was immediately stopped and ablation line displaced further.

### CT anatomy of the right phrenic nerve and merge with the electroanatomical mapping

Patients underwent pre-procedural gated 64-slices cardiac CT scan, with delineation of the course of the RPN by a radiologist with extensive expertise in cardiac imaging, either using direct visualization of the RPN or inferring the position of the RPB by visualizing the right pericardiophrenic artery when RPN could not be directly seen. A new CT imaging sequence was created containing the original CT images with the path of the RPN overwritten by a 5 pixel wide three-dimensional curved line at 900 HU value in attenuation to facilitate segmentation of the RPN. The cardiac chambers and PVs were also segmented. The segmented CT imaging was then merged with the electroanatomical mapping using a dedicated custom program on MATLAB software (Mathworks, Natick, MA). The shortest 3D distance between the CT-segmented RPN and every endocardial pace-mapping site was measured. Of note, the RPN segmentation was not made available at the time of AF ablation, and as such, the operator was blinded to the true RPN location.

### Statistical analysis

Categorical variables were presented as percentages and continuous variables as mean and standard deviation. Groups were compared by a parametric *t*-test. Correlation between distance and RPN threshold was performed using the Spearman’s *Rho* coefficient. Receiver operating characteristic (ROC) curve was performed for assessing the ability of the RPN threshold for predicting a distance to the RPN > 10 mm, which appears as a clinically pertinent distance for ensuring safe RF ablation.^14^ Sensitivity and specificity of each RPN threshold cut-off were also assessed. Statistical significance was defined by a *P* < 0.05.

## Results

In 45 patients, 1033 pace-mapping sites in proximity to typical course of the RPN were analysed (23 ± 16.4 pacing sites per patient). Clinical characteristics of the patients are summarized in *Table 1*.

**Table 1:** Clinical and procedural characteristics of study patients

	*n* = 45
Age (y)	61.9 ± 10.1
Female gender	14 (31.1%)
Hypertension	20 (44.5%)
Diabetes mellitus	5 (11.1%)
Dyslipidaemia	21 (46.7%)
History of stroke	9 (20%)
CHA_2_DS_2_-VASc score	1.9 ± 1.6
Beta-blocker	30 (66.7%)
Class I antiarrhythmic drugs	13 (28.9%)
Class III antiarrhythmic drugs	18 (40%)
Direct oral anticoagulants	32 (71.1%)
Vit-K antagonists	13 (28.9%)
Paroxysmal AF	41 (91.1%)
Left atrial size (cm)	4.3 ± 0.7
LVEF (%)	59 ± 6
Procedural duration (min)	172.7 ± 103.3
Fluoroscopy duration (min)	48.5 ± 23.5
X-ray exposure (µgy·m^2^)	1209 ± 875
RPN mapping in LA	45 (100%)
RPN mapping in RA	6 (13.3%)
Number of RPN pace-mapping points per patient	23 ± 16.4

All patients underwent pace-mapping of the RPN on the septal portion of the right PVs in the LA (872 pacing sites), and 6/45 (13.3%) also underwent RPN pace-mapping on the lateral/posterior part of the SVC and RA (161 pacing sites). Amongst the 1033 pace-mapping sites analysed, 725 (70.2%) were RPN capture sites including capture threshold assessment: 74 sites (7.2%) with capture threshold ≤ 10 mA, 319 sites (30.1%) with capture threshold > 10 mA and ≤20 mA, 135 sites (13.1%) with capture threshold > 20 mA and ≤30 mA, and 197 sites (19.1%) with capture threshold > 30 mA and ≤50 mA. The remaining 308 sites (29.8%) were in proximity to capture sites but demonstrated non-capture of the RPN at 50 mA.

**Figure 1:**
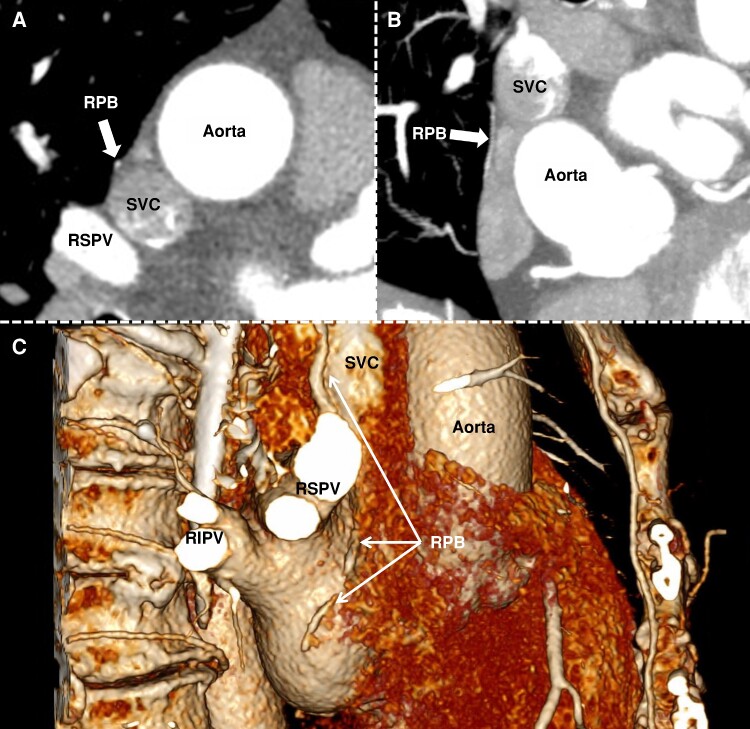
Delineation of the pericardiac RPB course using CT scan. *Panel A* shows an axial slice at the level of the RSPV, and *Panel B* an adjusted coronal slice, with the RPB highlighted (white arrow). *Panel C* shows a right lateral view of the 3D reconstruction of the CT scan, with the pericardiac course of the RPB highlighted (white arrows). RIPV, right inferior pulmonary vein; RPB, right pericardiophrenic bundle; RSPV, right superior pulmonary vein; SVC, superior vena cava.

**Figure 2:**
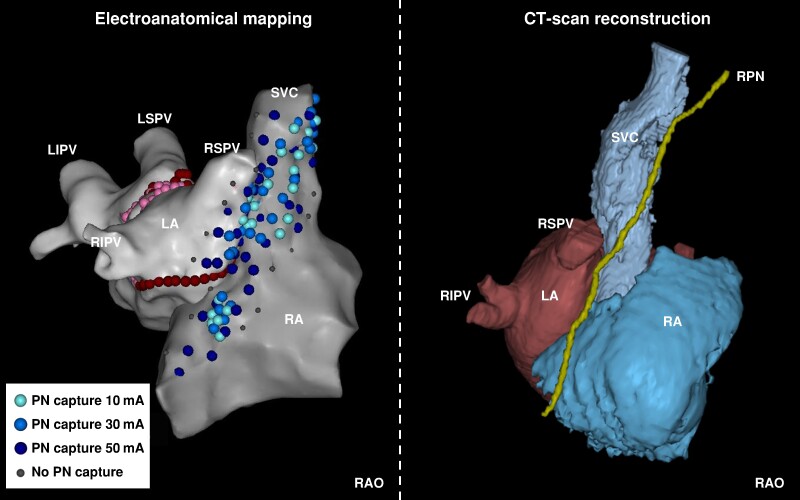
Left panel: electroanatomical mapping of the right and left atrium with RPN pace-mapping sites, colour-labelled with the RPN capture threshold. Right panel: corresponding 3D reconstruction of the CT scan, with the course of the RPN segmented in yellow. LA, left atrium; LIPV, left inferior pulmonary vein; LSPV, left superior pulmonary vein; RA, right atrium; RIPV, right inferior pulmonary vein; RPN, right phrenic nerve; RSPV, right superior pulmonary vein; SVC, superior vena cava.

**Figure 3:**
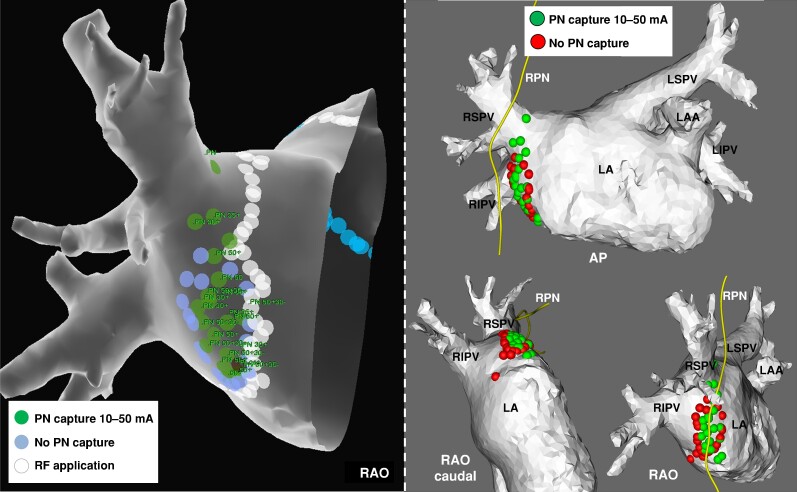
Left panel: electroanatomical mapping of the septal portion of the right pulmonary veins ostium, showing RPN pace-mapping sites labelled with the capture threshold. Right panel: 3D reconstruction of the CT scan, with merging of the pace-mapping sites, using MATLAB software. The RPN is segmented in yellow. For facilitating visualization of capture and non-capture sites, all capture sites are displayed in green, regardless of the threshold. LA, left atrium; LAA, left atrial appendage; LIPV, left inferior pulmonary vein; LSPV, left superior pulmonary vein; RA, right atrium; RIPV, right inferior pulmonary vein; RPN, right phrenic nerve; RSPV, right superior pulmonary vein; SVC, superior vena cava.

The delineation of the course of the RPN was feasible in all patients in CT scan (*Figure 1*), allowing for the computation of the distance between the location of the RPN and pace-mapping sites. *Figures 2* and *3* show the visual correlation between pace-mapping sites and RPN reconstruction using CT scan, in two patients. The CT scan documented a proximity to the RPN from the LA endocardium that varied from 1 mm to >3 cm in the region typically anticipated to be within proximity to RPN. Of particular note, 34 of 872 pacing sites (3.9%) performed on LA septum were within 3 mm to the RPN and 302 (34.6%) were within 10 mm to the RPN.

**Figure 4:**
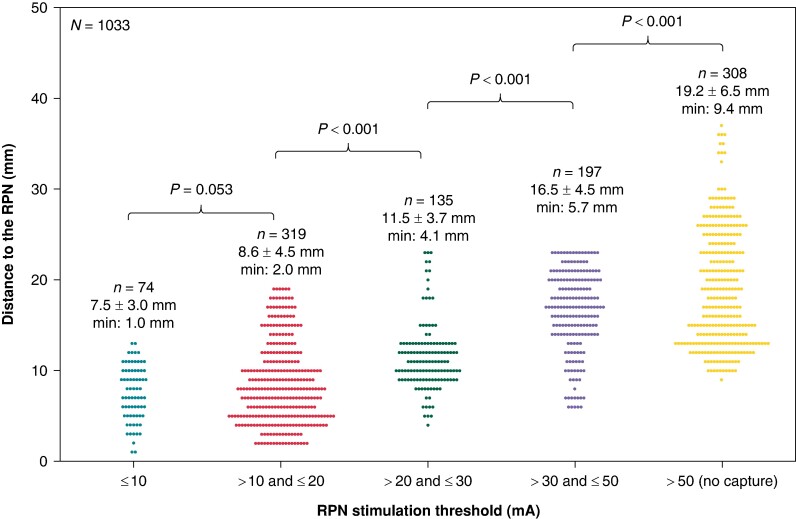
Distance between endocardial pace-mapping sites and the RPN, depending on the capture threshold.

There was a strong linear correlation between pacing threshold and distance to the RPN (*rho* = 0.692, *P* < 0.001). The distances from pacing sites to RPN were 7.5 ± 3 mm (min: 1 mm) when threshold was ≤10 mA, 8.6 ± 4.5 mm (min: 2 mm) when threshold was >10 and ≤20 mA (*P* = 0.053 for comparison), 11.5 ± 3.7 mm (min: 4.1 mm) when threshold was >20 and ≤30 mA (*P* < 0.001), 16.5 ± 4.5 mm (min: 5.7 mm) when threshold was >30 and ≤50 mA (*P* < 0.001), and 19.2 ± 6.5 mm (min: 9.4 mm) when there was no capture at 50 mA (*P* < 0.001; *Figure 4*).

**Figure 5:**
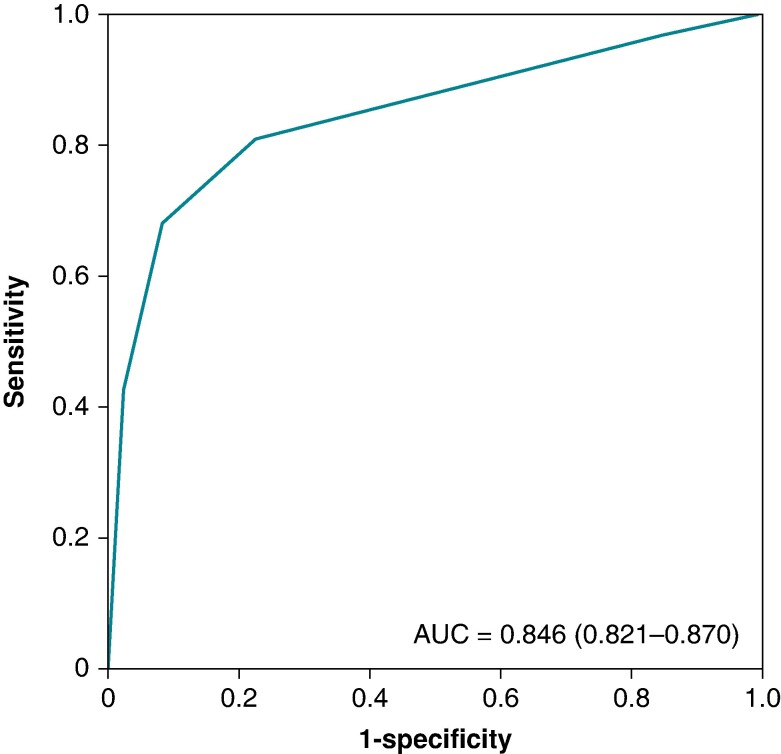
ROC curve assessing the value of the RPN capture threshold for predicting a distance to the RPN > 10 mm.

Right phrenic nerve pacing threshold predicted a distance > 10 mm to the RPN with an area under the ROC curve of 0.846 [0.821–0.870] (*P* < 0.001; *Figure 5*). *Table 2* summarizes the sensitivities and specificities of each threshold cut-off for predicting a distance > 10 mm to the RPN. A RPN capture threshold > 10 mA had a high sensitivity (96.8%) but a very low specificity of 15%, implying that 85% of the endocardial sites closer than 10 mm from the RPN demonstrated a RPN capture threshold > 10 mA. The specificity rose to 77.5% for RPN threshold > 20 mA, and 91.6% for RPN threshold > 30 mA. The highest specificity was reached with non-capture at 50 mA with a value of 97.6%, at the price of a low sensitivity (42.4%).

**Table 2:** Sensitivities and specificities of the different RPN capture threshold values for predicting a distance > 10 mm to the RPN

RPN pacing threshold for predicting distance to the RPN > 10 mm	Sensitivity	Specificity
>10 mA	0.968	0.150
>20 mA	0.808	0.775
>30 mA	0.680	0.916
>50 mA (no capture)	0.424	0.976

Finally, no RPN palsy was demonstrated in the study patients by avoiding energy application at sites with RPN capture at ≤ 50 mA.

## Discussion

### Main results and implications for safe RF ablation

This study addresses a gap in knowledge regarding the actual distance between the atrial endocardium and the RPN in the setting of RPN pace-mapping during AF ablation. By utilizing CT segmentation, we integrated anatomical details of the RPN with atrial electroanatomical mapping, offering a comprehensive understanding of the RPN’s relationship to the LA endocardium and appropriate pacing sites, for identifying sites at risk of RPN injury.

We confirmed that the size of the virtual electrode capturing the RPN is dependent on pacing output, and accordingly, that RPN’s capture threshold is linearly correlated with the distance between endocardial pacing sites and the anatomic location of the RPN. The distances ranged from 7.5 ± 3.0 mm when RPN capture threshold was ≤10 mA, to 19.2 ± 6.5 mm when there was no capture at 50 mA.

Based on the previous study by Nakagawa *et al*.,^14^ we chose a 10 mm distance cut-off between pacing site and RPN location as being clinically pertinent to ensure safety, since RF lesions on beating hearts at moderate, high, or very-high power induce maximal lesions depth of 6.7 ± 0.5 mm and maximal lesion diameter of 12.0 ± 0.6 mm (and thus, a lesion radius of 6 mm).

We found that RPN pacing threshold was able to predict a distance > 10 mm with an area under the ROC curve of 0.846 [0.821–0.870]. However, no specific threshold value had both excellent sensitivity and specificity, because of important distance overlap between different threshold categories. Potential reasons for this overlap include the non-spherical but rather dog-bone shaped morphology of the virtual cathode during pacing,^7,15^ the variable fatty characteristics of the tissue surrounding the RPN that could act as stimulation barriers, and finally integration errors between CT scan and electroanatomical mapping.

Nevertheless, the very high specificity observed with non-capture at 50 mA/2 ms (97.6%) suggests its potential as a reliable indicator for ensuring safe RF ablation. While the sensitivity at this threshold is lower (42.4%), the emphasis on specificity is crucial in the clinical context to avoid RPN injury. This low sensitivity may result in one displacing planned antral isolation ablation lines further outside the PV ostium than necessary to ensure a safe distance to the RPN, but this is usually achievable without being particularly challenging. On the other hand, it is important to acknowledge that electrophysiology stimulators limited to 20 mA appear inadequate for RPN pace-mapping. In fact, the absence of RPN capture at 20 mA may be falsely reassuring due to a specificity of only 77.5%, implying that almost a fourth of pacing sites closer than 10 mm from the RPN will show no RPN capture at 20 mA, and therefore there might be a risk of RPN injury.

Although much less frequent than during cryoballoon ablation of AF, RPN palsy is also an established potential complication of RF ablation,^3,4^ with classical symptoms being varying degrees of dyspnoea, cough, or hiccups. While the majority of patients fully or partially recovers RPN function within a year, some patients keep long-term debilitating symptoms. Multiples modalities are currently used for mitigating the risk of RPN injury, such as upstream pacing of the RPN in the SVC while the diaphragmatic function is monitored by right hemidiaphragm movement or compound motor action potentials,^16,17^ direct imaging of the RPN by pre-procedural CT,^18,19^ and pace-mapping of the RPN for avoiding RF application at capture sites.^20^ However, most studies assessing pace-mapping of the RPN utilized electrophysiology generators limited to 20 mA, which is insufficient for providing guaranteed safety according to our study results.

The recent study by Liu *et al.*^21^ used per-procedural intracardiac echocardiography for localizing the RPN during AF ablation procedures, and compared distances to the RPN from 88 capture sites depending on the pacing threshold, using a maximal pacing output of 20 mA with a 2 ms pulse width. However, there was no analysis of non-capture sites. They found that endocardial sites demonstrating an RPN capture threshold between 10 and 20 mA were 2.6–8.1 mm distant from the RPN, which aligns with our findings and further indicate that a higher output should be used for RPN pace-mapping.

Besides cryoballoon ablation, other thermal single-shot devices such as the RF balloon^22^ and the laser balloon^23^ both carry potential risk for RPN injury during right PV ablation. Therefore, high-output pace-mapping of the RPN might also be of interest in these cases for avoiding as much as possible energy delivery at sites demonstrating phrenic nerve capture, but this remains to be evaluated in further studies.

### Additional implications of proximity of the phrenic nerve on CT scan to LA endocardium

Much to our surprise the phrenic nerve appears to be ≤3 mm of the endocardial surface of the left atrium in ∼4% of pacing sites. This is closer than anticipated and may help explain the uncommon occurrence of phrenic nerve palsy reported with pulsed field ablation (PFA).^24^

Nerves are more resilient to PFA than myocardium and have a much higher irreversible electroporation threshold,^25^ likely due to the protection of the fatty low-conductivity myelin sheath absorbing the majority of the voltage drop. However, since the magnitude of the electric field delivered to the RPN is directly dependent on its distance to the ablation catheter,^26^ it is probable that the electric field delivered to closely located RPN can reach high enough levels for inducing—at least—reversible electroporation, as temporary RPN palsy has been demonstrated in humans or animals following PFA.^24,27,28^ Furthermore, PFA generates some Joule heating from the delivery of electric fields and resulting current flow through a resistive tissue load. As such, thermal heating may still occur with PFA for several mm surrounding the electrode surface^29,30^ and may also explain this observed unanticipated complication. Perhaps pacing threshold determination with right PV isolation before PFA application may also help to eliminate this uncommon complication if minor adjustment of pulsed field energy location can minimize proximity when very low threshold for capture is observed.

### Limitations

This is a single centre study involving a relatively limited number of patients; however, this is counter-balanced by the large number of pace-mapping sites. The delineation of the RPN or RPB using CT scan can sometimes be difficult, which could potentially lead to identification errors. We used integration between CT scan imaging and electroanatomical mapping for measurements of the distances, and despite all efforts made and the experience of the physicians involved, merging errors between the two imaging modalities remain possible.

## Conclusion

This study underscores the interest of high-output pace-mapping of the RPN during AF RF ablation. The correlation between pacing threshold and the distance to the phrenic nerve provides valuable insights for clinicians aiming to mitigate the risk of phrenic nerve injury during ablation procedures. Non-capture at 50 mA/2 ms emerges as a highly specific indicator for predicting a distance > 10 mm to the phrenic nerve, supporting its utility in ensuring safe RF delivery.

## Data Availability

Data will be made available upon reasonable request.
